# The Effect of Potassium Nitrate Supplementation on the Force and Properties of *Extensor digitorum longus* (EDL) Muscles in Mice

**DOI:** 10.3390/nu15061489

**Published:** 2023-03-20

**Authors:** Tomas Liubertas, Jonas Liudas Poderys, Vilma Zigmantaite, Pranas Viskelis, Audrius Kucinskas, Ramune Grigaleviciute, Jonas Jurevicius, Dalia Urbonaviciene

**Affiliations:** 1 Department of Coaching Science, Lithuanian Sports University, 44221 Kaunas, Lithuania; 2 Biological Research Centre, Lithuanian University of Health Science, 47181 Kaunas, Lithuania; 3 Institute of Horticulture, Lithuanian Research Centre for Agriculture and Forestry, 54333 Babtai, Lithuania; 4 Institute of Cardiology, Membrane Biophysics Laboratory, Lithuanian University of Health Sciences, 50162 Kaunas, Lithuania

**Keywords:** nitrates, nitric oxide, potassium nitrate, BALB/c mouse model, muscle histology, muscle force, athletic performance, sarcopenia, muscle recovery

## Abstract

Adding potassium nitrate (KNO_3_) to the diet improves the physiological properties of mammalian muscles (rebuilds weakened muscle, improves structure and functionality). The aim of this study was to investigate the effect of KNO_3_ supplementation in a mouse model. BALB/c mice were fed a KNO_3_ diet for three weeks, followed by a normal diet without nitrates. After the feeding period, the *Extensor digitorum longus* (EDL) muscle was evaluated ex vivo for contraction force and fatigue. To evaluate the possible pathological changes, the histology of EDL tissues was performed in control and KNO_3_-fed groups after 21 days. The histological analysis showed an absence of negative effects in EDL muscles. We also analyzed 15 biochemical blood parameters. After 21 days of KNO_3_ supplementation, the EDL mass was, on average, 13% larger in the experimental group compared to the controls (*p* < 0.05). The muscle-specific force increased by 38% in comparison with the control group (*p* < 0.05). The results indicate that KNO_3_ has effects in an experimental mouse model, showing nitrate-diet-induced muscle strength. This study contributes to a better understanding of the molecular changes in muscles following nutritional intervention and may help develop strategies and products designated to treat muscle-related issues.

## 1. Introduction

The effects of (NO_3_^−^) and nitric oxide (NO) on the muscle circulatory system and mitochondrial and contractile efficacy [1,2] may increase muscle blood circulation and improve the metabolic response to physical activity [2]. The evidence indicates that even the concentration of plasma nitrites is an independent factor of physical performance [3,4]. Nevertheless, studies on the effects of nitrate (NO_3_^−^) on performance ability have been highly controversial so far [5,6,7,8,9,10,11].

Recently beetroot became popular and actively researched (in various forms) as the dominant source of dietary NO_3_^−^, primarily due to its vast availability and the simple form of preparation suitable for final consumption. Remarkably, limited studies have evaluated alternative NO_3_^−^-rich leafy vegetables and even fewer sports-related studies have focused on inorganic nitrates. To our knowledge, this is the first comprehensive reported evaluation of the effects potassium-nitrate-solution (instead of sodium nitrate) supplementation on muscle power.

Historically, a class of inorganic nitrates has been related to multiple health hazards. However, at the same time, an extensive number of inorganic nitrate compounds contain sodium nitrate (NaNO_3_) and potassium nitrate (KNO_3_) (labeled E251 and E252, respectively), and are used as food additives and have been approved by the European Food Safety Authority [5], the US [6], and Australia and New Zealand [7].

The presumption that dietary nitrates could be of harm to human health is debatable, keeping in mind that >80% of all nitrates discovered come from vegetables, the product group that is known for its beneficial action on human health [11]. There are two prevalent known methods of obtaining NO: metabolizing NO endogenously and synthesizing dietary nitrates via the (NO_3_^−^)/nitrite (NO_2_^−^) and nitric oxide (NO) pathway from consumed food products [12]. The link of inorganic dietary nitrate consumption to possible health benefits for the prevention of hypertension and other forms of cardiovascular disease has been widely researched [13,14,15]. Interestingly, some scientific works have outlined the importance of oral bacteria playing a significant role in nitrate bioactivation [16,17,18].

Nitric oxide is a free radical that lacks a paired electron. Until the recent discovery of NO as a signaling molecule, it was believed that most free radicals bear a negative effect on macromolecules such as DNA, proteins, and lipids [19,20]. NO is a relatively stable free radical that reacts only with metals and other free radicals [21].

Multiple academic works have confirmed high NO bioavailability not only as vasodilator, i.e., lowering blood pressure [22,23,24], inhibiting platelet aggregation [16,25,26], improving blood vessel functions [27], and increasing mitochondrial capacity [1], but also influencing brain functions and taking part in immune responses [28,29]. NO that is synthetized in the blood-vessel endothelium regulates blood pressure and bloodstream velocity and, after being released from central nervous system (CNS) neurons, influences the traveling of nervous impulses and takes part in cognitive and memory functions [29,30].

Potassium is well known as an important ion in the human body and is associated with multiple physiologic and pathophysiologic processes [31]. Potassium is particularly important for protein and carbohydrate circulation, cell-membrane processes, nerve and muscle excitability, and nerve-impulse transmission. Potassium deficiency has been strongly associated with an increase in arterial blood pressure. A number of studies comparing levels of potassium have proven that potassium supplementation has shown to reduce arterial hypertension and is particularly useful in individuals with a history of arterial hypertension. Physical activity and training may result in an increase in intracellular potassium concentrations in resting muscles and relatively lower plasma potassium concentrations compared to values reported in untrained individuals. Additionally, a blunting of the exercise-induced hyperkalemia in trained individuals is associated with a decrease in the net loss of K^+^ from contracting muscles; these observations have been attributed to an upregulation of the Na^+^−K^+^ pump activity in both inactive tissues and active muscles [32].

Mild potassium deficiency causes general weakness and fatigue, muscle weakness, twitching, cramps, pain, an increased heart rate, excessive urination, and worsening of diabetes control [33]. The effects of potassium intake on coronary-heart-disease-prevention serves as a rationale for athletic dietary recommendations. High dietary potassium is associated with a decrease in blood pressure, particularly in the context of a high-sodium, also referred to as “western”, diet.

To assess whether KNO_3_ supplementation had any effect on working muscles, only young mice were used in this trial to eliminate any factors and conditions related to aging. In parallel, this study assessed whether KNO_3_ causes destructive processes within the muscles. To the best of our knowledge, this is the first comprehensive reported evaluation of the study with a hypothesis that the use of potassium-nitrate solutions instead of sodium-nitrate supplementation could have a positive synergistic effect: K^+^ and NO_3_^−^. Our findings show that KNO_3_ has effects in an experimental mouse model, showing nitrate-diet-induced muscle strength. Most earlier studies analyzing the effects of nitrates on physiological and functional properties were based on dietary nitrates or inorganic nitrates in the form of sodium nitrate, but very few analyzed health-related aspects of potassium nitrate. Health benefits demonstrated by the two elements separately (K^+^ and NO_3_^−^) makes potassium nitrate a considerably very competitive supplemental material in athletic performance and wellness in general. In this study, we hypothesized that the use of a KNO_3_ diet would improve the physiological properties of mammalian muscles (rebuilding weakened muscle, improving their structure and functionality) with further application in humans. Therefore, this study was conducted to clarify the effect of a potassium nitrate diet on skeletal muscles’ (EDL) maximum and specific force, and other parameters. In addition, in order to analyze biochemical adaptation to the potassium-nitrate supplementation, this study investigated biochemical blood parameters and the histology of EDL.

## 2. Materials and Methods

All research involving animals was conducted according to the requirements of the European Commission directive and the permit to perform procedures (No. G2-172) from the Lithuanian ethics commission at the State Food and Veterinary Service Animal welfare department.

### 2.1. In Vivo Experimental Model

BALB/c mice (*Mus musculus*) (8–12 weeks old, 22.1 ± 2.87 g, *n* = 21) were acquired from the Lithuanian University of Health Sciences, Academy of Veterinary, Vivarium. All animals were kept under the standard hygiene conditions in the Biological Research Centre. All laboratory animals were kept in equal conditions in specially equipped cages. The groups were separated (control and experimental) and fed food specially designed for rodents ad libitum with a 12:12 h day/night cycle and a noise level not exceeding 85 db. Supplementation was administrated for 21 days. The experiment was approved by State Food and Veterinary Service, 7 February 2017, permission No. G. 2-56.

All animals were divided into two groups depending on the diet type (control and KNO_3_-fed groups). Both groups were fed a standard rodent diet; the KNO_3_-fed group was supplemented with the tested substance via drinking water—the water was available at all times. Water bottles (150 mL) were checked daily, and the intake between groups was compared during refill (every day). The KNO_3_-fed group’s water bottles were supplemented with a KNO_3_ water solution. An amount of 2 g potassium nitrate dissolved in 1000 mL of water was later distributed to the drinking containers of the experimental group. After complete dissolution, KNO_3_ did not change the properties of the drinking water to a noticeable extent—no drinking water consumption difference was observed between the groups. The water supply was not interrupted. Average water consumption per mouse was assumed to be 10 mL per day, equivalent to 20 mg of KNO_3_ to 25 g of mouse body mass (1.25 mg per 1 g of body weight). Assuming a standard consumption of 10 mL of water per day, containing 2% potassium-nitrate concentration, the daily intake resulted in 10 mg KNO_3_ per day. No force feeding was used in order to exclude any stress-related factors that could distort the results of this experiment.

The animals were checked daily; agility and health status were evaluated. Once a week, the animals were evaluated by two investigators using blind testing—investigators were not aware of the group differences. The general health of the laboratory animals was evaluated according to Burkholder et al. [34].

After 21 days (3 weeks), the animals were sedated with carbon dioxide and euthanized by cervical dislocation. Once death was verified, blood samples and the EDL muscles were removed for further analysis.

### 2.2. Histology of Tissues and Blood Sample Collection

Blood samples were collected by cardiac puncture in Microvette^®^ (Sarstedt AG & Co. KG, Nümbrecht, Germany) 200 tubes with EDTA anticoagulant (BD, Vacutainer, Winnersh, UK). Following the selected method, muscle power was measured [35]. Organs were placed in the fixation solution, Formalin 10% (buffer) (Sigma Aldrich, St. Louis, MO, USA). The samples were then prepared for histological analysis using Anthony’s procedure [36]; histological preparations were performed at the LUHS Pathology Center and evaluated by veterinarian pathologists. Histological analysis was performed on the *Extensor digitorum longus* muscle (EDL). The muscle was taken after isometric-force measurement and fixated into formalin solution using the technique by Meyerhoff and colleagues [37].

Fixation: Small pieces of tissue were placed in solutions of chemicals that cross-link proteins and inactivate degradative enzymes, which preserves cell and tissue structure. Dehydration: The tissue was transferred through a series of increasingly concentrated alcohol solutions, ending with 100%, which removes all water. Clearing: Alcohol was removed in organic solvents in which both alcohol and paraffin are miscible. Infiltration: The tissue was then placed in melted paraffin until it became completely infiltrated with this substance. Embedding: The paraffin-infiltrated tissue was placed in a small mold with melted paraffin and allowed to harden. Trimming: The resulting paraffin block was trimmed to expose the tissue for sectioning (slicing) on a microtome. Paraffin sections were cut at 14 μm thickness for light microscopy. The sections were placed on glass slides and stained with a combination of hematoxylin and eosin (H&E). Hematoxylin stains DNA in the cell nucleus, RNA-rich portions of the cytoplasm, and the matrix of cartilage, producing a dark blue or purple color. In contrast, eosin stains other cytoplasmic structures and collagen pink [36].

### 2.3. Blood Samples Analysis

The peripheral blood samples from all animals with EDTA were used for blood morphological tests. Blood parameters measured for blood morphology: white blood cells (WBC; 10^9^/L), lymphocytes (LYM; % and 10^9^/L), monocytes (MON; % and 10^9^/L), granulocytes (GRA; % and 10^9^/L), red blood cells (RBC, 10^12^/L), hemoglobin (HGB, g/L), hematocrit (HCT, %), mean corpuscular volume (MCV, fL), mean corpuscular hemoglobin (MCH, pg), mean corpuscular hemoglobin concentration (MCHC, g/L), red cell distribution width (RDW, %), platelets (PLT, 10^9^/L), mean platelet volume (MPV, fL), procalcitonin (PCT, %) and platelet distribution width (PDW, %).

After a morphological blood test, the tubes were centrifuged (Eppendorf Centrifuge 5810R, Warsaw, Poland) at 5 min. 3000 rpm for the separation of blood plasma. The blood plasma was used for the creatine kinase determination (CK). CK tests were carried out using an automated computerized biochemical analyzer SELECTRA Junior (Vital Scientific, Spankeren, The Netherlands), using Spinreact reagents (Girona, Spain).

### 2.4. EDL Muscle-Force Measurement

The EDL muscle performance was measured by ex vivo muscle-contraction experimentation. The maximum force of the muscle was calculated by Lab Chart Power Lab software v.8.1.24 and Power Lab acquisition hardware (AD Instruments, Colorado Springs, CO, USA) according to the methodology [37].

The specific force of muscles was calculated according to the formula:

*sP*_0_ (kN/m^2^) = *P*_0_ × (*m*/*L_f_* × *k*) (1)

where *sP*_0_—specific force (kN/m^2^); *P*_0_—maximum absolute isometric force kN/m^2^; *m*—muscle weight (mg); *L_f_*—the production of optimum fiber length (mm); and *k*—muscle coefficient or the density of mammalian muscle (1.06 mg/mm^3^).

### 2.5. Statistical Analysis

The data were expressed as a mean value and standard deviations. Statistical comparison between the groups was performed using two-way analysis of variance (ANOVA) with the Wilkinson–Mann–Whitney test, nested ANOVA with the Levene test, and post hoc analysis with Tukey’s test. SPSS ver. 22.0 (SPSS Inc., Chicago, IL, USA) was used for calculations. Effect size was found using Cohen’s d_z_ statistical calculation for paired samples. Differences were significant at *p* < 0.05.

## 3. Results

### 3.1. Effects of Dietary KNO_3_ on Body Weight

All 21 experimental animals survived three experimental weeks. No behavior changes were noticed.

During the experimental period, no significant increase in body weight was recorded (Table 1). However, there were significant differences between the EDL/body weight ratios of the control and experimental (KNO_3_ fed) groups (Table 1). The ratio of EDL (mg) and EDL (mm) to body weight increased, respectively, by 43% and 34%. (Table 1). The photograph of the EDL muscle extirpate from the mice can be found in the Supplementary Materials (Figure S1).

**Figure 1:**
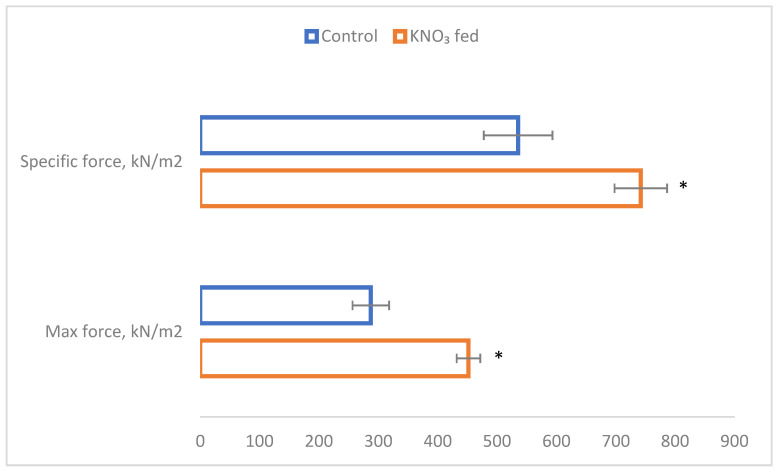
The specific and maximum force of EDL muscles of control and KNO_3_-fed mice. Significant differences are indicated by asterisks (*).

To provide a quantifiable measure of muscle activation, we used the maximum absolute isometric force and the production of optimum fiber length to calculate specific muscle force (*sP*_0_). Our analysis showed that *sP*_0_ was significantly higher in KNO_3_-fed mouse muscles, resulting in a greater muscle physiological parameter (*p* < 0.05) compared to the controls (Figure 1). There were no significant weight differences between the groups after 21 days (Table 1).

Nitrate supplementation in mice influenced EDL function. After 21 days, in mice that were fed nitrate, the EDL weight was, on average, 13% larger than in controls (*p* < 0.037), with a Cohen’s d_z_ of 0.47. After 21 days of potassium-nitrate supplementation, the muscle-specific force increased by 38% compared to the control group (*p* < 0.029), with a Cohen’s d_z_ of 0.54. (Figure 1).

### 3.2. Histology of the Tissues

We also performed the histopathology of organs from both groups to investigate the impact of KNO_3_. After 21 days of supplementation, organs of the sacrificed mice, including EDL muscles after isometric-force measurement, were fixed with formalin. Histological images of selected organs were taken under light microscope. The histology of the EDL muscle samples was examined for structural differences, comparing the control and experimental groups. It can be assumed that the changes were not detected in the muscles of the control group; however, supplementation of potassium nitrate in the experimental group (KNO_3_-fed group) resulted in muscles that were heavier and bigger in size.

**Figure 2:**
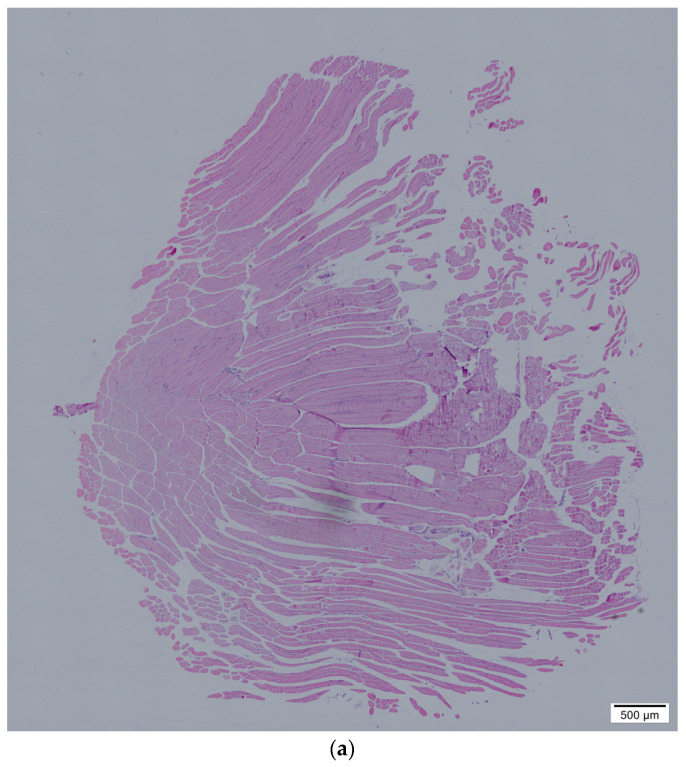

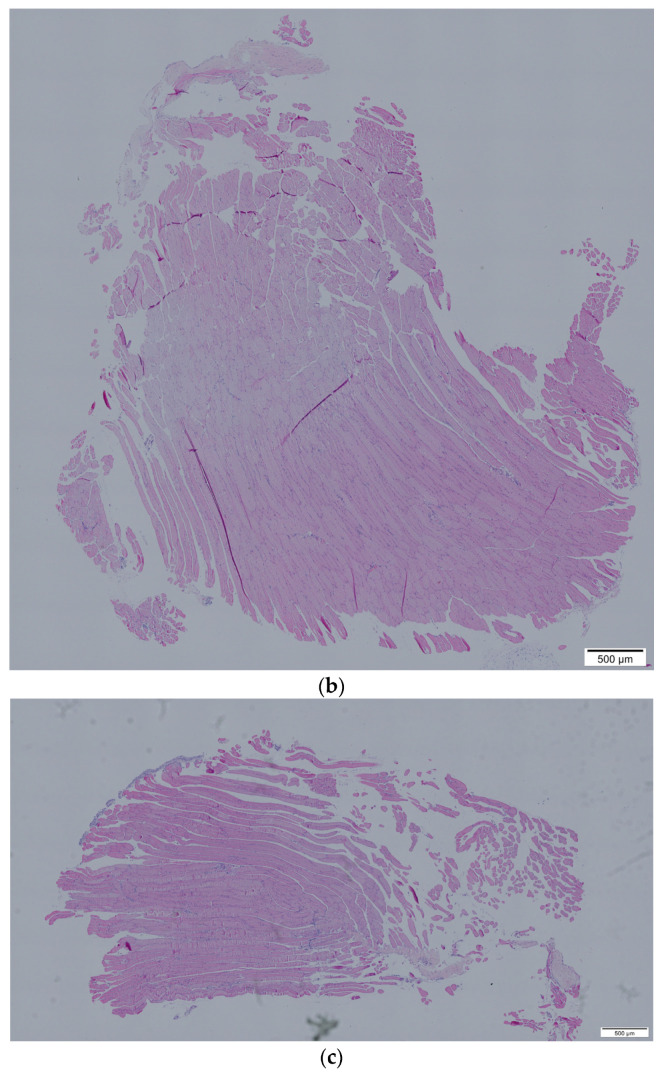

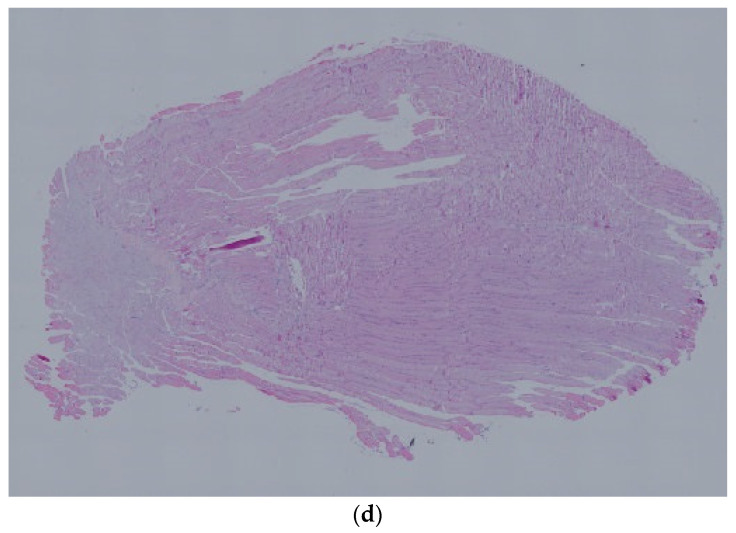
Histological imaging of formalin-fixed KNO_3_-fed mouse EDL part tissue slices 21 days after treatment. (**a**,**c**)—control group; (**b**,**d**)—KNO_3_-fed group. The magnified histological experiment staining images are in the Supplementary Materials (Figure S2).

A microscopic photograph shows the EDL muscle’s structural differences comparing control and experimental mice (Figure 2). We include the possibility that some muscle fibers of EDL muscles could have been broken down during the treatment period within the control group. These muscles did not withstand even the 10 V measurement of isometric force on isolated mouse muscles in vitro. In the KNO_3_-fed group, all EDL treated muscles were able to withstand 20 V voltage. Assessing EDL muscle structure in the control group, we found that the muscles were structurally heterogeneous. Contrarily, in the KNO_3_-fed group, the muscle fibers of EDL muscles were homogeneous and more evenly distributed.

### 3.3. Effects of KNO_3_ on Blood Chemical Parameters

The blood chemical analysis results are presented in Table 2. Three weeks of supplementation with KNO_3_ resulted in significant differences in HGB and PLT between the mouse groups (Table 2). The HGB concentration increased 0,8 times in the KNO_3_-fed group compared to the controls, respectively, 177 and 140 × 10^9^/L. The normal mouse HGB ranges from 136 to 159 × 10^9^/L. The platelets (PLT, 10^9^/L), mean platelet volume (MPV, fL), procalcitonin (PCT, %), and platelet distribution width (PDW, %) also increased. The main function of platelets (PLT) is primary hemostasis, and platelet production can increase due to inflammatory processes. Nevertheless, in thrombocytosis, PLT should increase and MPV should decrease; in this case, however, both indicators were elevated.

**Figure 3:**
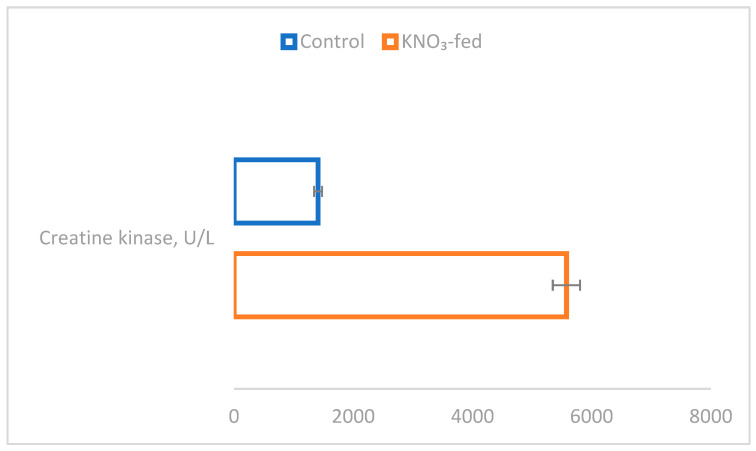
Creatine kinase indication.

The creatine kinase (CK) enzyme system is an important energy-delivering mechanism in skeletal and cardiac muscles. It is known that, under physiological conditions, functional requirements of muscles and cellular energy supply are well coordinated, but the mechanisms maintaining this balance are still unknown [38]. It is widely believed that cytoplasmic and mitochondrial CK are important to the intracellular transport of high-energy phosphate between (ATP) generating and utilizing sites within muscle cells during steady-state activity [39]. In this study, the blood CK biomarker increased by more than four times in the experimental group (Figure 3).

The histology of the EDL muscles showed an absence of inflammatory processes. It is assumed that KNO_3_-fed mice improved the intracellular transport of high-energy and muscle strength during ATP synthesis.

## 4. Discussion

The possible existence of cancerogenic properties related to nitrates [38,40] has created a negative attitude towards this unique compound, which has been in the academic spotlight for the past decades. Health-related hazards encouraged the creation of strict regulations regarding the amount of nitrates allowed in food products and drinkable water. The statement that nitrates could be harmful to our health is rather debatable, keeping in mind that >80% of all nitrates (referred to as dietary nitrates) that we consume come from vegetables—the product group that is known for its beneficial action on health [11,41]. Very few studies so far have given their attention to potassium nitrate (KNO_3_) as a possible source of nitric oxide [42,43]. There is a curious occurrence of nitric-oxide production in skeletal muscle in response to physical exercise. Nitric oxide is produced by NO synthase (eNOS) in the vascular endothelium and is also produced by NOS1 (neuronal NOS), NOS2 (inducible NOS), and NOS3 in skeletal muscle, and is synthesized from l-arginine. NOS1 and NOS3 are both expressed in skeletal muscle and can be increased by chronic exercise. NO interacts with the metabolic enzyme, AMPK [44]. NO and AMPK cooperatively regulate PGC1α [37] and 70–90% of NO is stored in S-nitrosothiols [45], the main source of NO in tissues. NO has a very intense metabolic action; it reduces lipid accumulation and inflammatory process in the liver, activates pancreatic blood perfusion and insulin secretion, enhances mitochondrial activity and glucose metabolism in the muscles, stimulates the conversion of white fat into brown fat in adipose tissue, and decreases the concentration of circulating triglycerides [46]. NO is a molecule with a half-life in the blood measurable in milliseconds. Early academic research showed that muscle weakness is a principal factor causing multiple disorders associated with movement disorders and increased mortality [47].

Given the technical properties of potassium nitrate—comparatively neutral flavor (compared to sodium nitrate and other “natural” sources), comparatively smaller quantity it is necessary to consume in order to reach the recommended concentration (compared to extracts and juices), ease of use (solubility, small volume), availability and standardization (food-grade availability) and already-established food safety regulation protocol—the added results of our research suggest KNO_3_ as a seriously competing compound in the field of sports nutrition, wellness, and muscle-related disease prevention, etc.

The two separate elements (K^+^ and NO_3_^−^) demonstrated multiple health benefits, which gives us a strong scientific base to raise a hypothesis that the use of a potassium-nitrate solution instead of sodium nitrate, or alternative dietary sources of nitrate in daily supplementation, could have positive synergistic effect. The aim of our research was to evaluate the occurring pathological changes in EDL tissues along with properties resulting in structural changes (occurrence of possible negative formations) and changes in muscle force, mass, and corresponding blood parameters. The histology was performed in control and KNO_3_^−^-fed groups after 21 days of supplementation. The histological analysis of previously mentioned tissues showed the absence of negative factors in mouse EDL muscles. After the 21-day feeding period, the *Extensor digitorum longus* (EDL) muscle was evaluated ex vivo. The EDL muscle is typically used for contractility measurements, as this muscle is relatively easily dissected with intact tendons for the purpose of attachment to a force transducer. In mice, the EDL is dominated by type-2 fibers and is used as a model for studies on fast-twitch-muscle function [48]. The histology of the EDL tissues of both the control and KNO_3_^−^-fed groups was performed to address the safety of KNO_3_ supplementation. Our study shows (based on histology data) no negative KNO_3_ effect in mouse EDL muscles. This study also analyzed 15 biochemical blood parameters.

Significant differences in EDL force parameters were found between control and KNO_3_-fed groups. After 21 days of supplementation with potassium nitrate, the EDL mass was, on average, 13% larger than that of the non-supplemented controls (*p* < 0.037). After 21 days of potassium-nitrate supplementation, the muscle-specific force increased by 38% in comparison with the control group (*p* < 0.029). The denominator standardizes the difference by transforming the absolute difference into standard deviation units. Cohen’s term d is an example of this type of effect size index. Cohen classified the effect sizes as small (d_z_ = 0.2), medium (d_z_ = 0.5), and large (d_z_ ≥ 0.8). The effect size was medium for muscle-specific force and EDL mass, respectively, with values of 0.54 and 0.47. The effect of KNO_3_ feeding on muscle histology was also observed in the biochemical composition of the blood.

The results of our study indicate that KNO_3_ has effects in an experimental mouse model of nitrate-diet-induced muscle strength. The histological analysis of this study reported no muscle damage in the mice. However, elevated levels of serum CK could be a potential indicator of inflammatory processes elsewhere in the body. Elevated levels of CK usually observed in athletes as a postworkout hormonal response suggest a possible connection of muscle damage and elevated levels of CK. However, this study did not identify any muscle damage and, therefore, no relation to elevated levels of CK, suggesting an alternative point of view to the direct linkage of CK and muscle injury. Interestingly, the degree of muscle mass increase was greater than the increase in body weight. This suggests that KNO_3_^−^ possibly has a greater proportional effect on muscles than on other organ systems in mice. The KNO_3_-fed mice had significantly increased muscle-force production. This increase in muscle strength was proportional to the degree of increase in muscle mass and offers physiological evidence for a functional improvement in EDL muscles ex vivo. Recent studies in the field of KNO_3_ that analyze human and/or animal skeletal muscle focus mainly on exercise and performance (before and after exercise); however, none of the studies cover the topic of muscle changes related to non-exercise supplemental nutrition.

Different studies have examined the effect of nitrate supplementation in in vivo and in vitro models, raising the hypothesis of muscle-nitrate reserves that could be affected by nitrate ingestion [49,50]. Colleagues proposed alternative mechanisms involving Ca^2+^ by which dietary NO_3_^−^ may influence muscle-contractile function in humans; however, all of them agree that additional research is required in order to determine the exact mechanisms of the reaction. We believe that our research is an insightful addition to this specific field of study. The results indicate that KNO_3_ had effects in an experimental mouse model, showing nitrate-diet-induced muscle strength. This study, together with other similar studies, contributes to a better understanding of the molecular changes behind muscle physiology. These findings, together with the technical qualities of KNO_3_, may have implications for novel nutrition-based strategies for athletes for muscle-injury prevention and faster muscle recovery, and even suggests a blank mechanism of muscular changes related to non-exercise supplemental nutrition, which provides a strong base for future research with practical implications regarding the formulation of new athletic supplements, supplements related to the prevention of muscle loss, also known as sarcopenia, faster muscle recovery, etc. Moreover, it is important to note that this is the first study related to a “non-exercise” approach which also covers the possible negative aspects (cancer, tumor-related implications), providing future researchers and/or practitioners with additional ideas in the field of disease, trauma prevention/treatment, research related to movement restrictions (such as prolonged bed regime, space travel, etc.), or even an alternative view on the topic of muscle atrophy.

The limitations of this study are that it was partially restricted by the initial raised hypothesis, assuming that 21 days of supplementation would be too short a period for structural changes to take place; however, we believe that fiber typing should be included in future experimental designs in order to understand even the slightest dynamics of biochemical and structural deviation. Moreover, this study did not cover the possible fatigue and recovery properties of the EDL muscle in mice, which could have been altered by 21 days of KNO_3_ supplementation.

## 5. Conclusions

Our research results suggest that nitrate is necessary for the physiological function of muscles. The use of a potassium-nitrate solution in a mammalian diet demonstrated a positive symbiotic effect. The supplementation of potassium nitrate in a mammalian diet improved the physiological properties of muscles and demonstrated the potential to rebuild weakened muscle, improving its structure and functionality. After KNO_3_ supplementation for 21 days, the EDL mass was, on average, 13% larger in the experimental group than in the controls (*p* < 0.05). Since skeletal muscle uses its nitrate reservoir to support muscle physiology, it can be assumed that nitrate supplementation may be used to improve smooth-muscle contractile functions, especially in diseases associated with muscle weakness and/or loss. The muscle-specific force increased by 38% in comparison with the control group (*p* < 0.05). These results indicate that KNO_3_ has effects on the experimental mouse model, showing nitrate-diet-induced muscle strength. This is also interesting from a nutritional perspective since several leafy green vegetables are high in inorganic nitrate, and the amount of nitrate used in the present study can be easily achieved by adopting a ‘green’ diet. These results could provide a new mechanism by which nitrate demonstrates beneficial effects on muscle strength that can be applied in the field of competitive sports, therapeutic applications, trauma prevention, prolonged immobilization and related muscle atrophy, space-travel-related muscle weakening and deterioration, etc.

This study, together with other studies, contributes to a better understanding of the molecular changes behind many processes associated with muscle movement and will help researchers develop better strategies to improve muscle health, muscle-related-trauma prevention, and possibly contribute to the area of age-related muscle-loss research.

## Figures and Tables

**Table 1:** Body weight and EDL muscle: body weight ratio in control and KNO_3_-fed mice.

	Control (*n* = 6)	KNO_3_ (*n* = 15)
Body weight (g)	24.60 ± 2.41	23.10 ± 2.50
EDL (mg): body weight (g)	0.29 ± 0.01	0.51 ± 0.02 *
EDL (mm): body weight (g)	0.36 ± 0.01	0.55 ± 0.06 *
EDL muscle mass (mg)	7.70 ± 0.20	8.76 ± 0.32 *

*Note.* All data are expressed as standard error of means ± SD. Asterisks (*) within the same line indicate significant differences between the control and KNO_3_-fed group (*p* < 0.05).

**Table 2:** Effects of KNO_3_ on blood chemical parameters in a diet.

Parameter (Unit)	Control	KNO_3_-Fed
WBC (10^9^/L)	6.41 ± 1.72	7.54 ± 0.63 *
LYM (10^9^/L)	4.17 ± 0.91	5.64 ± 0.15
MON (10^9^/L)	0.97 ± 0.04	0.95 ± 0.01
GRA (10^9^/L)	1.2 ± 0.05	0.96 ± 0.02
RBC (10^12^/L)	6.99 ± 0.59	8.72 ± 0.21 *
HGB (g/L)	140.80 ± 9.93	177.21 ± 5.02 *
HCT (%)	0.34 ± 0.03	0.43 ± 0.01 *
MCV (fL)	40.48 ± 0.38	49.39 ± 0.30 *
MCG (pg)	16.87 ± 0.28	20.31 ± 0.24 *
MCHC (g/L)	349.01 ± 4.53	411.43 ± 5.10 *
RDWc (%)	13.55 ± 0.64	16.05 ± 0.13 *
PLT (10^9^/L)	253.77 ± 26.45	390.29 ± 29.49 *
MPV (fL)	0.18 ± 0.01	0.31 ± 0.04 *
PCT (%)	6.07 ± 0.72	7.83 ± 0.45 *
PDWc (%)	14.67 ± 1.70	18.48 ± 0.15 *

*Note.* All data are expressed as standard error of means ± SD. Asterisks (*) within the same line indicate significant differences between the control and KNO_3_-fed group (*p* < 0.05).

## Data Availability

Data are contained within the article and the Supplementary Materials.

## Supplementary Materials

The following supporting information can be downloaded at: https://www.mdpi.com/article/10.3390/nu15061489/s1, Figure S1: The photograph of the EDL muscles extirpate from mic; Figure S2: Histological imaging of formalin-fixed mouse EDL part tissue with myofibers and its myonucleis slices in 21 day fed of KNO_3_ study after treatment. (A)—control group; (B)—KNO_3_ fed group.
