# Supplementary material for: The Effect of Potassium Nitrate Supplementation on the Force and Properties of Extensor digitorum longus (EDL) Muscles in Mice
Source: Nutrients. 2023 Mar 20;15(6):1489. doi: 10.3390/nu15061489 (PMC10057731; doi:10.3390/nu15061489)
Supplement: Supplementary file 1 [file nutrients-15-01489-s001.zip › nutrients-2265846-supplementary.pdf]

Supplementary material:

The Effect of Potassium Nitrate Supplementation on the Force and Properties of Extensor Digitorum Longus (EDL) Muscles in Mice

Tomas Liubertas [1,*], Jonas Liudas Poderys [1], Vilma Zigmantaite [2], Pranas Viskelis [3], Audrius Kucinskas [2], Ramune Grigaleviciute [2], Jonas Jurevičius [4] and Dalia Urbonaviciene [3]

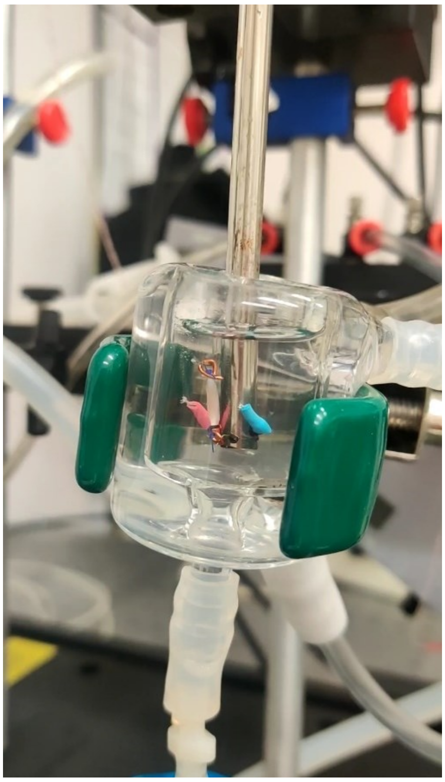

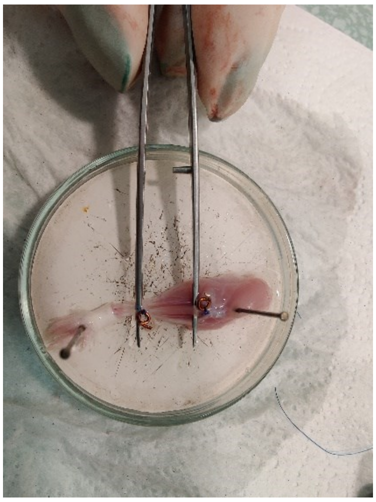

Figure S1. The photograph of the EDL muscles extirpate from mice

A)

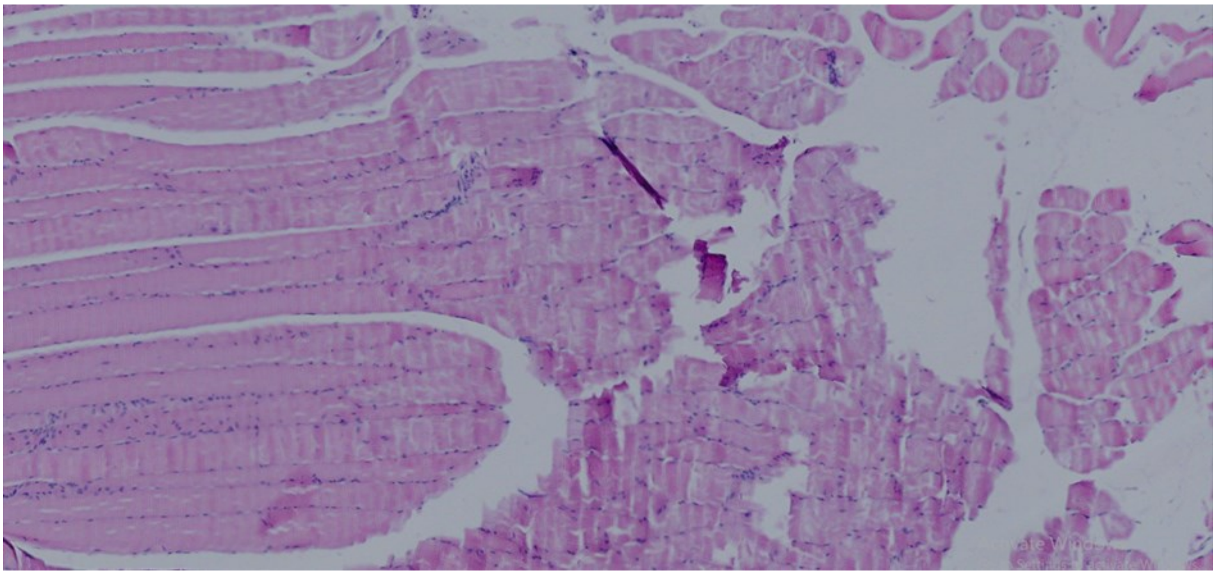

B)

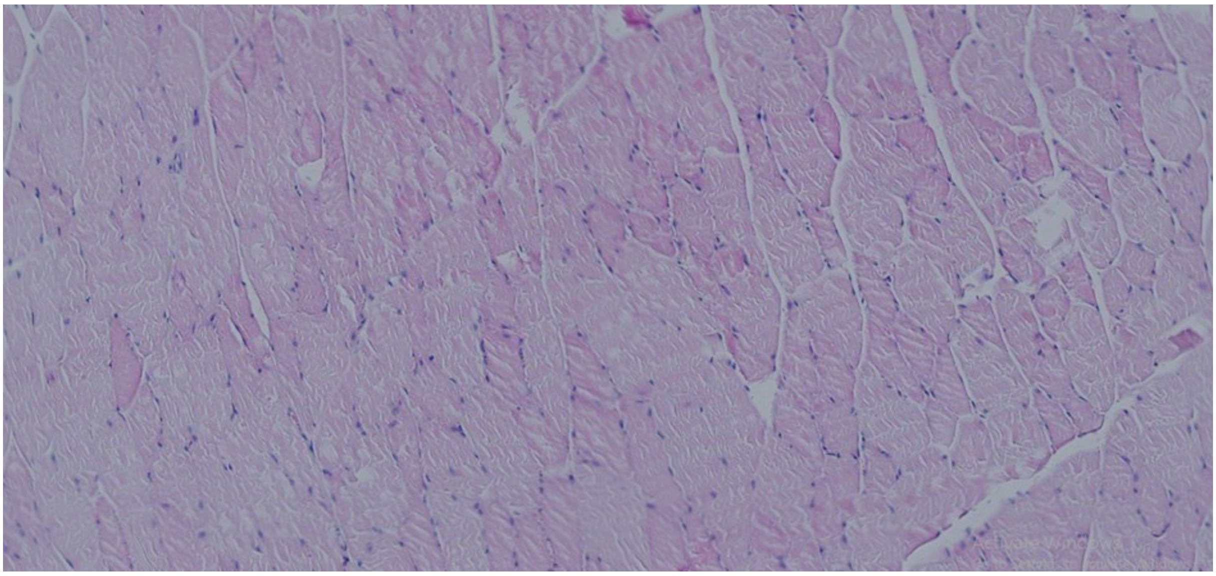

Figure S2. Histological imaging of formalin-fixed mouse EDL part tissue with myofibers and its myonucleis slices in 21 day fed of KNO_3 study after treatment. (A) – control group; (B) – KNO_3 fed group.
